# Finite element analysis of impacted canine disimpaction: effects of anchorage systems and T-loop gable bend angles

**DOI:** 10.3389/fbioe.2026.1850735

**Published:** 2026-06-05

**Authors:** Xingyu Li, Xibei Li, Shaoyang Yu, Yingyue Zhang, Wenke Yang, Jun Guo, Xueyao Jiang, Xiao Yan, Qiang Zhang, Xiao Yuan

**Affiliations:** 1 Department of Orthodontics, The Affiliated Hospital of Qingdao University, Qingdao, China; 2 School of Stomatology, Qingdao University, Qingdao, China

**Keywords:** canine impaction, finite element analysis, mini-implant, orthodontic, T-loop, transpalatal arch

## Abstract

**Introduction:**

To compare the effects of micro-implant (MI) and transpalatal arch (TPA) combined with T-loops of varying gable bend angles on the distalization of mesially impacted canines.

**Methods:**

A finite element model of a mesially impacted left maxillary canine was developed using patient CBCT data. Separate models combining MI or TPA with T-loops featuring α-end angulations of 0°, 15°, and 30° were created. A 1 N retraction force was applied, and canine displacement, stress distribution, and rotation were analyzed.

**Results:**

Increasing the α-end angulation from 0° to 30° changed canine movement from uncontrolled tipping (crown distal/root mesial) to controlled translation (concurrent crown–root distal displacement). The 30°angulation achieved the best root control, minimizing crown–root displacement differences along the X-axis. Among the tested conditions, the 30° angulation showed the most favorable root control pattern in this analysis. Greater angulation also produced lingual root torque and intrusion, maintaining root position within the alveolar bone. Both anchorage systems enabled distalization; however, the TPA group showed slightly greater canine displacement with minor mesial movement and extrusion of anchorage teeth.

**Conclusion:**

Increasing the T-loop α-end angulation converts uncontrolled tipping to controlled translation by synchronizing crown and root displacement. Angulations of 15°–30°are recommended for optimal root control. While MI offers greater anchorage stability, TPA remains a practical noninvasive alternative despite some anchorage loss.

## Introduction

The impaction rate of canines ranges from 2% to 3% in the general population and accounts for approximately one-fourth of all tooth impactions (Cooke, 2006). Permanent maxillary canine transposition, which refers to the positional exchange of two adjacent teeth within the same dental quadrant, represents a particularly challenging condition associated with canine impaction (Bishara, 1992; Shapira and Kuftinec, 1989).

Given the essential functional role of canines in the dental arch, several interventions are typically undertaken when impaction occurs. The use of continuous archwire mechanics—regardless of bracket type—can produce undesirable side effects on adjacent teeth. In contrast, with the segmented arch technique (SAT), after stabilizing the anchorage unit, targeted canine movement can be achieved using cantilevers or loops (Caldas et al., 2014).

The T-loop remains a safe and effective design that continues to serve as the predominant choice for tooth retraction and space closure (Cai, 2020; Chacko et al., 2018; Rose et al., 2009). Previous studies have reported the use of mini-implant (MI) or transpalatal arch (TPA) in combination with T-loops for the distal movement of mesially impacted canines. Ozkan et al. employed two types of anchorage devices with different force-activation mechanisms to achieve successful distalization of mesially displaced canines (Ozkan and Bayram, 2016). Similarly, Ferreira et al. retracted ectopic canines using a TPA combined with a modified T-loop (Ferreira et al., 2019). The intraosseous positional changes of transposed canines depend on the magnitude, direction, and point of force application (Rodriguez-Cardenas et al., 2021). By adjusting the gable bend angle, the T-loop can facilitate canine distal movement and root control by generating an appropriate moment-to-force (M/F) ratio (Braun and Garcia, 2002). In a case involving a buccally impacted canine overlapping the lateral incisor, Hsu et al. (2016) successfully employed MI combined with T-loops featuring gable bends to resolve the impaction.

Finite element analysis (FEA) has long been utilized in *in vitro* orthodontic biomechanics research (Wu et al., 2025; Zhang et al., 2023). Previous studies have demonstrated that torque control of the anterior teeth can be effectively and efficiently achieved using closing loops with varying degrees of gable bend tailored to the treatment plan (Anh et al., 2021). However, the biomechanics of canine distal movement using T-loops with different gable bend angles remain unclear, and the influence of various anchorage devices on canines and anchorage teeth requires further investigation.

This study aimed to simulate orthodontic tooth movement during the activation of a 1 N T-loop using the finite element (FE) method and to compare the effects of MI and TPA, each combined with T-loops of varying gable bend angles, on the distal movement of canines.

## Materials and methods

Cone-beam computed tomography (CBCT) data were obtained from a 13-year-old female patient presenting with bilateral impacted canines. The inclusion criteria were as follows (Cooke, 2006): unilateral or bilateral mesially impacted canines (Bishara, 1992); eruption of all other permanent teeth except the canines (Shapira and Kuftinec, 1989); absence of appreciable root resorption in the remaining teeth (Caldas et al., 2014); normal development of tooth root morphology and length; and (Cai, 2020) absence of periodontal disease. Prior to the commencement of the study, the participant and her legal guardian provided written informed consent. Ethical approval for the study was obtained from the Ethics Committee of the Affiliated Hospital of Qingdao University.

**FIGURE 1:**
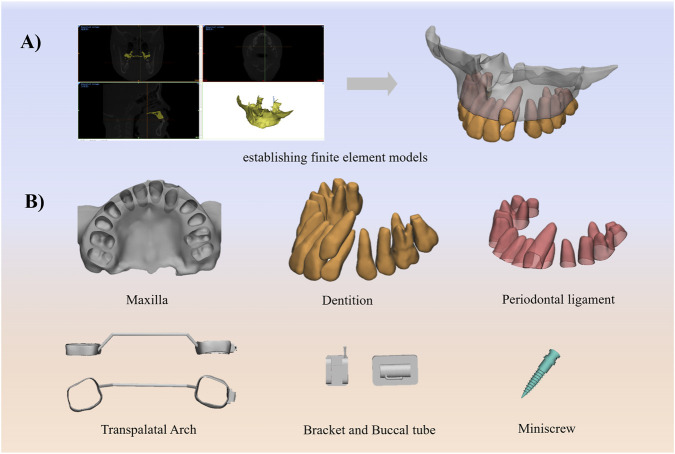
Three-dimensional finite element models. **(A)** The maxilla and maxillary dentition were reconstructed based on the CBCT data. **(B)** The maxillary tissues were modeled using patient data, while the brackets, buccal tubes, orthodontic bands, TPA, and MI were constructed according to available commercial products.

The CBCT images were obtained using a Carestream CS9300 unit (Carestream Health, United States) with the following parameters: 85 kV, 4 mA, an exposure time of 11.26 s, and a dose of 175 mGy·cm^2^. The CBCT data were imported into the three-dimensional (3D) image processing software Mimics 21.0 (Materialise, Belgium) to generate a preliminary 3D model through thresholding (Figure 1). Subsequently, Geomagic Wrap 2015 (Geomagic, United States) was used for surface refinement, noise reduction, and smoothing, resulting in a smooth, continuous, and closed 3D tooth model. Following remeshing, the right completely impacted canine and the retained deciduous tooth were removed from the model. The periodontal ligament (PDL) was modeled in 3-matic 12.0 (Materialise, Belgium) by offsetting the roots of all teeth by 0.25 mm (Wang et al., 2024). The archwires, brackets, bands, TPA, and MI components were created in Unigraphics NX (UG, Germany) and subsequently imported into 3-matic for assembly with the teeth and maxilla.

The T-loop was fabricated from 0.018 × 0.025-inch (0.46 × 0.64 mm) titanium–molybdenum alloy (TMA) archwires, featuring an anterior gable bend at an angle of 0°, 15°, or 30° (Mo et al., 2020) relative to and positioned distal to the canine bracket (Figure 1). The mesial and distal arms of the T-loop were designed at different vertical levels to generate biologically optimal extrusion forces. The bracket and buccal tube slots measured 0.022 × 0.028 inch (0.56 mm × 0.71 mm).

**FIGURE 2:**
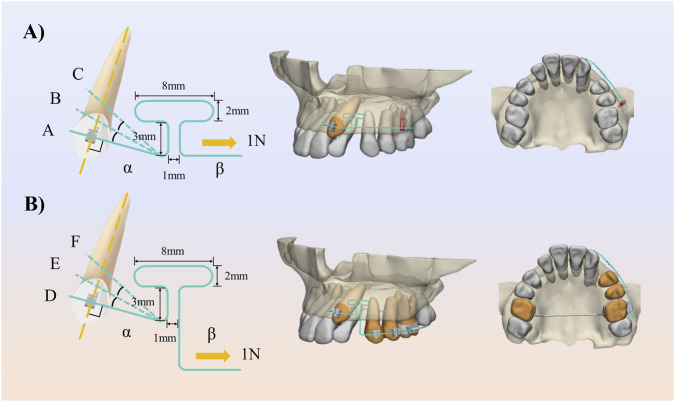
Six main groups. **(A)** MI Group: Groups A–C used micro-implant (MI) for anchorage with alpha-end angulations of 0°, 15°, and 30°. **(B)** TPA Group: Groups D–F used transpalatal arch (TPA) for anchorage with alpha-end angulations of 0°, 15°, and 30°.

In the NX software, all components were precisely aligned, and six 3D models were constructed (Figure 2). In groups A, B, and C, an MI measuring 2 mm in diameter and 10 mm in length (Figure 2A) was placed apically between the roots of the maxillary second premolars and first molars, in combination with T-loops incorporating 0°, 15°, and 30° gable bends, respectively. In groups D, E, and F, T-loops with the same gable bend angles were inserted into the bracket slot of the maxillary canine at the anterior portion, while the posterior portion was inserted into the auxiliary slot of the molar buccal tube through the premolar bracket. The buccal tubes were welded to the molar bands, and a TPA connected the first molars bilaterally (Figure 2B).

A total of six simulation groups were established to investigate the biomechanical effects of different T-loop angulations on maxillary canine retraction using two distinct anchorage systems. All materials were assumed to be homogeneous and linearly elastic, and their mechanical properties (Young’s modulus and Poisson’s ratio) were adopted from previous studies (Table 1) (Wang et al., 2023). Each model underwent adaptive meshing in 3-matic to generate C3D4-type tetrahedral element meshes. MI group contained 737,402 elements and 189,466 nodes, while TPA group contained 803,980 elements and 211,724 nodes. Following mesh convergence verification (Supplementary Figure S1), each model underwent adaptive meshing in 3-matic with an average element size of 0.3 mm, producing C3D4 tetrahedral meshes. The MI group comprised 737,402 elements and 189,466 nodes, whereas the TPA group comprised 803,980 elements and 211,724 nodes.

**TABLE 1:** Material properties.

Material	Young’s modulus, E (MPa)	Poisson ratio, v
Teeth	19,600	0.30
Periodontal ligament (PDL)	0.69	0.45
Alveolar bone	13,700	0.30
Bracket/Buccal tube/orthodontic band/Transpalatal arch (TPA)	200,000	0.30
Mini-implant (MI)	114,000	0.34
Titanium–molybdenum alloy (TMA)	69,000	0.30

The application of force was divided into two stages. First, archwire deformation was simulated by applying a 1 N retraction force to the T-loop. For the different gable bend angles, reaction forces were calculated based on the displacement at the terminal position. The application of force was divided into two stages. First, archwire deformation was simulated by applying a 1 N distal traction force along the archwire to the β-end of the T-loop (Bou Malhab et al., 2024). Subsequently, for the different gable bend angles, reaction forces were calculated based on the displacement at the terminal position. No preactivation or anti-rotation bends were incorporated into the springs. To stabilize the model during force application, the maxillary base was designated as the fixed boundary region, and the displacement and rotational degrees of freedom of the maxilla were constrained in three-dimensional space. The teeth, brackets, PDL, and alveolar bone were defined as bonded, whereas the brackets and archwire, as well as adjacent teeth, were modeled in contact. Based on previous experimental data, a friction coefficient of μ = 0.15 was assumed to allow controlled sliding of the archwire along the bracket (Bou Malhab et al., 2024).

A global coordinate system was established using the mesioincisal angle point of the maxillary left central incisor, and a local coordinate system was defined for each tooth. In this coordinate system, the positive X, Y, and Z directions corresponded to the left, posterior, and apical directions, respectively. In the X (transverse) direction, a positive value indicated distal movement of the canine. In the Y (sagittal) direction, a positive value represented palatal movement, and in the Z (vertical) direction, a positive value denoted intrusion of the canine.

**FIGURE 3:**
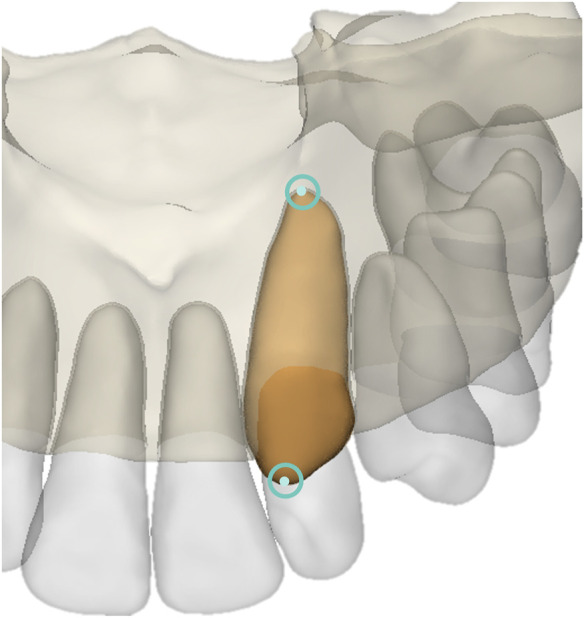
Locations of selected nodes. The reference points were identified based on their anatomical positions. For the canines, the most incisal tip and the root apex were selected as measurement nodes.

**FIGURE 4:**
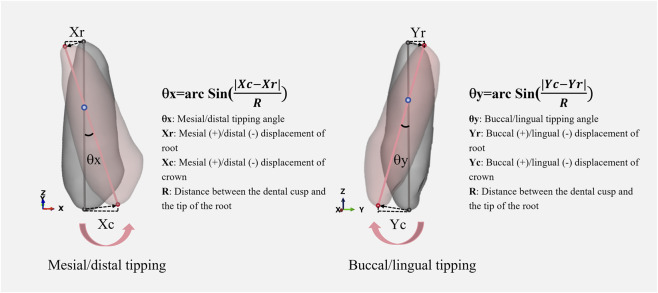
Tooth tipping movement indicators. Buccal/lingual and mesial/distal tipping angles were calculated based on the crown–root length and the displacements of the crown and root. Lingual and distal displacements were defined as positive values, whereas buccal and mesial displacements were defined as negative values. The principle underlying this angular calculation is that the displacement of two points within a specific plane determines the resultant angular change.

To quantify tooth displacement, reference nodes were positioned on the crown and root surfaces. The displacements of these landmark nodes along the X, Y, and Z-axes following orthodontic force application were analyzed using the finite element method (Figure 3). In addition to the three-dimensional displacements, tooth tipping movements were also calculated and included in the analysis. The tipping angles (buccal/lingual or mesial/distal) were determined based on the total tooth length and the displacements of both the crown and root (Figure 4) (Liu et al., 2023).

All FE analyses were performed using ABAQUS 2020 (Simulia, France). Von Mises stress was used to evaluate overall stress concentration. Positive minimum principal stress values represented tension, while negative values represented compression. Stress was measured in megapascals (MPa), and displacement in millimeters (mm). A color scale was used for visualization, with red indicating maximum values and blue indicating minimum values.

## Results

**FIGURE 5:**
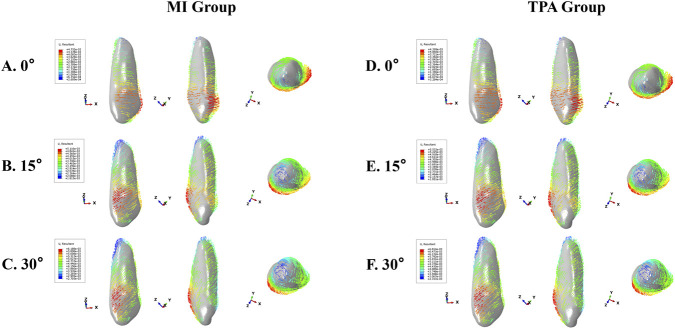
Three-dimensional visualization of the overall displacement of the left maxillary canine. **(A–C)** represent the overall displacement of the MI group, while **(D–F)** represent that of the TPA group. The color scale and vector lengths represent the magnitude of displacement, while the arrows indicate its direction.

Figure 5 and Table 2 illustrate the displacement results of the maxillary canine. The overall displacement patterns produced by T-loops of identical dimensions were similar, regardless of whether TPA or MI were used for anchorage.

**TABLE 2:** Displacements of the canine crown tip and root apex along the X-, Y-, and Z-axe.

Group		Angle	Displacements ( × 10^–4^) (mm)					
			X		Y		Z	
			Crown	Root	Crown	Root	Crown	Root
MI group	A	0°	2.5163	−0.3538	−0.205	−0.7753	0.2369	0.158
	B	15°	1.4924	0.6185	−2.189	0.4618	1.8204	1.0659
	C	30°	0.8899	0.8146	−2.7788	1.0941	2.7881	3.0236
TPA group	D	0°	2.6347	−0.2	−0.4838	−0.0579	0.3018	0.249
	E	15°	1.7314	0.6224	−2.4955	0.554	1.859	2.0188
	F	30°	1.2005	1.1062	−3.0333	1.1377	2.8064	3.0657

When a pure 1 N distal force was applied to the T-loop without a gable bend, comparison between Group A and Group D revealed that both anchorage systems produced total canine displacement characterized by distal tipping and distolingual rotation. Along the X-axis, the canine crown moved distally, while the root moved mesially in both groups. The crown displacement was considerably greater than the root displacement, indicating uncontrolled tipping. Along the Y-axis, the tooth exhibited distolingual rotation accompanied by labial displacement of both the crown and root, suggesting a potential tendency toward bony dehiscence. No significant extrusion or intrusion was observed along the Z-axis.

The introduction of a gable bend at the α-end produced noticeable changes in the three-dimensional displacement pattern of the canine. Slight differences were observed between the MI and TPA Groups, primarily reflected in the displacement of both the canine and anchorage teeth. Because the free segment of the archwire in the TPA Group was shorter than that in the MI Group, it exhibited greater rigidity. Under identical force conditions, this increased stiffness resulted in slightly greater canine displacement in all directions in the TPA Group compared with the MI group. Furthermore, since the TPA Group employed tooth-borne anchorage, mesial movement of the anchorage teeth was evident.

**FIGURE 6:**
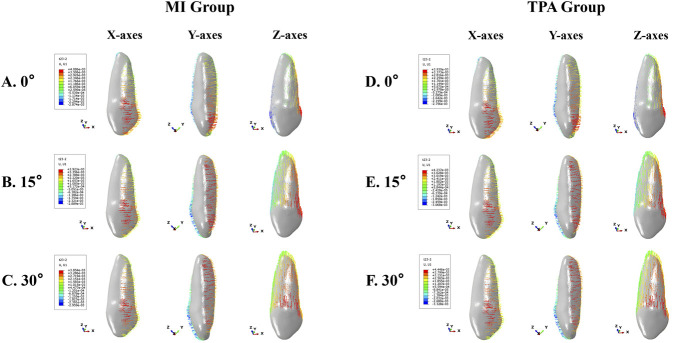
Displacement along the X, Y, and Z-axes of the left maxillary canine. **(A–C)** represent the three-dimensional directional displacement of the MI group, while **(D–F)** represent that of the TPA group.

As the gable bend angle increased, the overall magnitude of canine displacement also increased. The three-dimensional displacement maps revealed a progressively more distal and intrusive movement tendency in both the crown and root (Figure 6). Along the X-axis, the apical displacement gradually shifted from mesial to distal, moving in the same direction as the crown. This pattern indicates that the center of rotation moved apically toward the root apex, and tooth movement gradually transitioned from tipping to translation. Displacement data from Table 3 show that in both anchorage groups, increasing the gable bend angle reduced the difference in X-axis displacement between the crown and root—from initial values of 2.8701 × 10^−4^ mm and 2.8347 × 10^−4^ mm to 0.0752 × 10^−4^ mm and 0.0943 × 10^−4^ mm at 30°. The substantially decreased difference between crown and root displacement, together with the more uniform distal movement of both structures, demonstrates enhanced root control.

**TABLE 3:** Displacements between the crown and root along the X-, Y-, and Z-axes.

Group		Angle	Displacements ( × 10^–4^) (mm)		
			X	Y	Z
MI group	A	0°	2.8701	0.5702	0.0789
	B	15°	0.8739	−2.6508	0.7546
	C	30°	0.0752	−3.8729	−0.2355
TPA group	D	0°	2.8347	−0.4258	0.0528
	E	15°	1.109	−3.0495	−0.1599
	F	30°	0.0943	−4.1709	−0.2593

**FIGURE 7:**
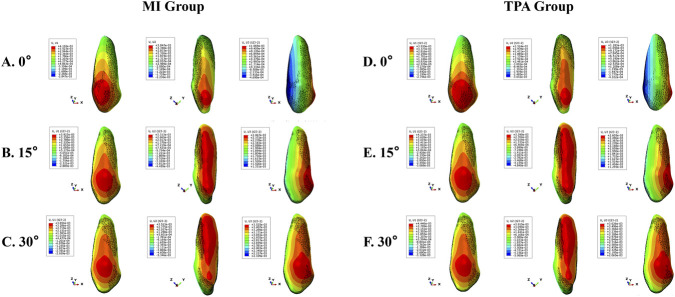
Tooth movement tendency along the X-, Y-, and Z-axes of the left maxillary canine in Models **(A–F)**. Comparison of pre- and post-displacement. The black mesh denotes the initial position, and the color map indicates the final position.

Along the Y-axis, a tendency toward apical palatal movement and coronal labial displacement was observed. This finding suggests that as the gable bend angulation increased, the risk of root apex perforation through the cortical bone decreased, which is favorable for tooth stability; however, vigilance should be maintained regarding the potential occurrence of bone fenestration. Three-dimensional evaluation revealed that the T-loop consistently induced distolingual rotation of the canine, although the position of the rotational fulcrum varied with the gable bend angulation. Overall, the fulcrum remained approximately aligned with the long axis of the tooth; however, as the angulation increased, it progressively shifted from a distal to a mesial position. The displacement superimposition diagrams revealed a consistent trend (Figure 7). To investigate the effect of the α-angle on tooth rotation during movement, angular measurements were calculated to reflect changes in the displacement pattern. Table 4 shows that although it did not comprehensively improve the 3D root control in tooth, they effectively reduced the crown distal tipping angle during distalization and increased the desirable lingual root torque.

**TABLE 4:** Tipping angles of the canine along the X- and Y-axes.

Variable	A	B	C	D	E	F
Mesial/distal tipping change (×10^–4^)	7.8047	2.3631	0.2033	7.7075	3.0001	0.2549
Buccal/lingual tipping change (×10^–4^)	1.5415	7.2019	10.5863	1.151	8.2993	11.422

In the comparison between MI and TPA Groups, beyond the slight differences in overall displacement magnitude, the movement patterns of the crown and root showed some variation. The difference in displacement between the crown and root reference points along the X-axis was greater in the TPA Group than in the MI group, indicating that the MI Group demonstrated superior root control in the mesiodistal direction.

**FIGURE 8:**
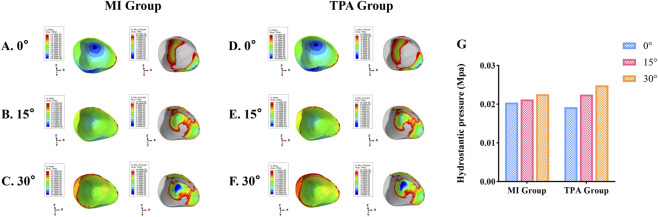
Von Mises and compressive stress distribution in the periodontal ligament (PDL) of the maxillary left canine. **(A–C)** represent the Von Mises and compressive stress distribution in the PDL of the MI group; **(D–F)** represent that of the TPA group; and **(G)** represents the changes in equivalent stress of the tooth under various loading conditions.

Analysis of stress distribution within the PDL of tooth left maxillary canine indicated that the von Mises stress increased with greater gable bend angulation. Higher stress concentrations were observed in the cervical regions on both mesial and distal surfaces. Regarding the maximum principal stress, compressive stress occurred in the distal cervical and mesial apical regions, whereas tensile stress was present in the mesial cervical and distal apical regions. At 0° in the MI group, the compressive stress zones were located in the mesial aspect of the apical region and in the distal cervical portion of the PDL (Table 5). As the angulation increased, the apical compressive stress zone gradually shifted distally and intensified, indicating apical displacement in the distal and apical directions and further confirming the root control effect (Figure 8).

**TABLE 5:** The maximum compressive stress in the periodontal ligament at the cervical and apical regions.

Variable	A	B	C	D	E	F
Cervical regions (MPa)	−0.0426	−0.0423	−0.0441	−0.0402	−0.0456	−0.0491
Apical regions (MPa)	−0.0111	−0.0215	−0.0335	−0.0124	−0.0229	−0.0338

**FIGURE 9:**
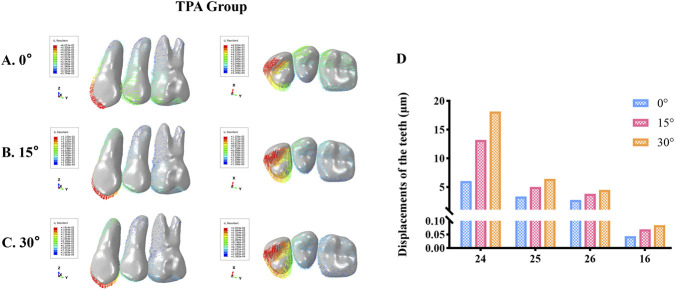
Displacement of anchorage teeth in the TPA group. **(A–C)** illustrate the displacement of the left maxillary anchor teeth in the TPA group at 0°, 15°, and 30°. **(D)** shows the overall displacement magnitude of each anchor tooth in the TPA group.

Although the different anchorage devices did not alter the overall canine displacement pattern, the displacement of the anchorage teeth in the TPA Group warranted attention. In Groups D, E, and F at 0°, the anchorage teeth—teeth 24, 25, and 26—exhibited mesial displacement, with tooth 24 showing the most pronounced movement accompanied by a slight intrusive tendency. Tooth 16, which was connected to tooth 26 via the TPA, showed a mild distal displacement tendency. As the gable bend angulation increased, the mesial movement and extrusive tendency of teeth 24, 25, and 26 became more evident (Figure 9).

## Discussion

For ectopically positioned canines, safely and efficiently guiding the tooth into its correct position is a primary objective during orthodontic treatment planning. The FE method, introduced to dental biomechanics research in 1973, enables non-destructive quantification of the stress distribution within the periodontium (Cattaneo and Cornelis, 2021).

**FIGURE 10:**
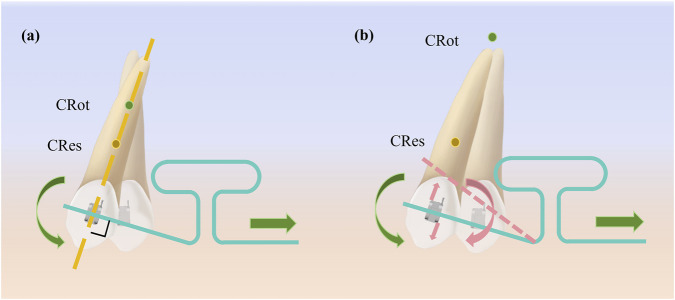
Force analysis of the left maxillary canine. **(a)** Uncontrolled tipping produced by a force bypassing the center of resistance (Cres), with the center of rotation (Crot) displaced apically. **(b)** The moment of force is represented in green, and the moment of torque is represented in pink.

Loops or springs are commonly used to facilitate the disimpaction of impacted teeth. Operating under the principle of frictionless mechanics, they provide greater anchorage control compared with friction-based mechanics. In the present study, two simulation models were constructed, each incorporating T-loops with three distinct α-end angulations. The three-dimensional displacement of the impacted maxillary canine and the stress distribution within its periodontal ligament were quantified (Figure 10). Increasing the α-end angulation of the T-loop enhanced root control, resulting in more effective and physiologically favorable canine distalization.

Biomechanical analysis of the canine revealed that applying a distal force not passing through the center of resistance is biomechanically equivalent to generating a force couple combined with a force traversing the center of resistance. Under these conditions, the center of rotation lies between the center of resistance and the root apex, resulting in opposite directional displacement of the crown and root. Biomechanical analysis shows that when a distally directed force is applied to the labial surface of the canine without passing through its center of resistance, the center of rotation lies between the center of resistance and the root apex. In the X-axis view, the crown moves distally and the root moves mesially; in the Y-axis view, both crown and root move labially. Under this condition, the M/F ratio is low, and the maximum stress within the PDL is concentrated at the distal apical region and the alveolar crest—this corresponds to the α = 0° scenario in the present study. Increasing the α-angle torque bend of the T-loop essentially introduces an additional couple into the wire-bracket system, generating an active root-distolingual torque of the wire within the bracket slot. As the α angle increases, the counter-couple produced by the wire increases, partially counteracting the mesial root tipping moment. Consequently, the net M/F ratio gradually increases toward the ideal range required for bodily translation, and the center of rotation shifts apically (Figure 10). In the X-axis, the mesiodistal inclination decreases, and the crown and root tend to move synchronously and in the same direction. In the Y-axis, increasing the α angle produces a more pronounced buccolingual rotation, manifesting as a positive torque pattern characterized by lingual root movement and labial crown tipping. And in the vertical dimension, the tooth exhibits a tendency toward intrusion.

Further analysis focused on the rotational angles of the tooth. The results showed that as the α-end angulation increased from 0° to 30°, the rotational angle around the X-axis approached zero, indicating increasingly parallel movement between the crown and root. This apical shift of the center of rotation reflects enhanced root control. Along the Y-axis, increasing α-end angulation produced more pronounced buccolingual rotation, exhibiting a positive torque pattern characterized by lingual root movement and labial crown inclination.

This study demonstrates that employing T-loops alone for canine distalization can result in undesirable distal crown tipping accompanied by mesial root movement. Clinically, the desired outcome is the repositioning of the apex of the transposed canine into its proper anatomical location. However, such distalization may also induce slight labial displacement of both the crown and root, increasing the risk of alveolar bone dehiscence and potentially compromising the long-term stability of the tooth. Introducing angulation at the α-end progressively reduced the discrepancy between the crown and root displacements of the canine along the X-axis. The initially opposite directions of crown and root movement became coincident, indicating improved root control. This mechanism is biomechanically analogous to the application of gable bends in closing loops or T-loops during space closure, which serve to prevent unwanted lingual inclination of anterior teeth. However, as reported in the literature, even when gable bends are increased to 60° or 90°, positioning the loop distal to the canine still limits controlled incisor movement—whether bodily translation or root movement (Jinnai et al., 2023). However, for a single tooth, when the α-angle is 30°, the crown-root displacement difference becomes minimal and is almost sufficient to achieve crown-root translation. The literature indicates that even increasing the gable bend to 60° or 90° still limits controlled incisor movement, whether bodily translation or root movement (Jinnai et al., 2023). However, for a single tooth, adding an appropriate gable bend during clinical follow-up appointments still helps to produce a tendency toward approximate bodily movement.

It is crucial to note that this finding is derived from finite element simulations. In clinical practice, the incorporation of angulation must be determined based on the actual situation, as increasing the angulation sacrifices the overall displacement of the tooth along the X-axis, potentially slowing the rate of distalization. Moreover, the increased stress at the root apex and alveolar crest may adversely affect both the treatment duration and root health.

From the perspective of the Y-axis, increasing angulation produced pronounced labial crown torque accompanied by lingual root torque. Although this torque effect is advantageous for maintaining the root apex within the alveolar bone boundaries, caution is warranted to mitigate potential risks of buccal bone dehiscence and gingival recession. Along the Z-axis, greater angulation resulted in increased apical displacement of both the crown and root, thereby helping to maintain the canine in a low position and ensuring that the root apex remained within the alveolar bone. Adequate surgical exposure and precise bracket positioning are essential prerequisites for effective traction, and a high position of the impacted canine within the vestibular fornix provides significant clinical advantages. Meanwhile, from the perspective of the Y-axis and Z-axis, the gable bend helps to maintain the canine in a low position and ensures that the root apex remains within the alveolar bone. A high position of the impacted canine within the vestibular fornix provides significant clinical advantages. In cases of canine transposition, tooth movement should ideally occur within the central portion of the alveolar ridge and traverse areas with the greatest width of attached gingiva (Ferreira et al., 2019). Consequently, during canine distalization, it is crucial to prevent root displacement beyond the alveolar housing and to avoid tooth extrusion. As shown in Figure 7, gable bends of 15° and 30° generated intrusive forces, with greater vertical displacement (intrusion) observed at larger bend angles. However, this study found that increasing the gable bend sacrifices the overall tooth displacement along the X-axis, potentially slowing the rate of distal movement and thus prolonging the traction duration and reducing traction efficiency. In addition, although the gable bend is beneficial for root control movement, a larger angle further increases compressive stress on the root, especially in the apical region. In addition, although the gable bend is beneficial for root control movement, a larger angle further increases compressive stress on the root, especially in the apical region. When the α angle is increased to 30°, the apical compressive stress in the MI and TPA groups rises from initial values of 0.0111 MPa and 0.0124 MPa, respectively, to about 0.0335 MPa and 0.0338 MPa. The markedly increased apical compressive stress may elevate the risk of root resorption and have a negative impact on pulp health. A constant 1 N distal traction force was applied to the archwire in this study. This force magnitude was selected based not only on previous finite element studies on impacted tooth traction, but also because conventional orthodontic forces typically range from 0.10 to 2 N, within which 1 N represents a moderate force generated by an archwire spring or loop, consistent with clinically routine force application. Furthermore, minor variations in the force magnitude do not significantly affect the core conclusions of this study, namely, that as the α angle increases, tooth movement transitions from tipping toward bodily translation.

The biomechanical system analyzed in this study did not include all teeth and therefore corresponds to the SAT. The SAT is widely employed in the management of impacted canine traction, molar uprighting, and other clinical applications due to its capacity to produce efficient biomechanical control. Segmented arch mechanics permit extended force application with constant low-load deflection, directional force consistency, and enhanced clinical efficiency, all of which contribute to the long-term stability of dental segments (Burstone, 1962). The unbonded maxillary left lateral incisor acted as a free body, allowing unobstructed clearance for the canine’s distal path. During distal movement, the canine root passing labial to the lateral incisor root induced physiological distopalatal crown tipping of the adjacent tooth. This “free-body effect” reduced the risk of root resorption compared with full-archwire engagement (Hsu et al., 2016).

This study employed two distinct anchorage devices. In this study, two different anchorage devices were employed, and the movement trends of the canine were generally similar between the two models. At each gable bend angulation, the canine displacement in the TPA group was consistently greater than that in the MI group, which is attributed to the difference in free archwire segment length (Hsu et al., 2016). In the MI group, the T-loop was inserted directly into the micro-implant, producing a longer effective free wire segment. In contrast, in the TPA group, the T-loop was placed anterior to the first premolar, shortening the free segment length. According to beam theory, the bending stiffness of a wire is inversely proportional to the cube of its length. Therefore, under the same activation angulation, the TPA group exhibited substantially higher stiffness and a steeper load-deflection curve. However, because of anchorage loss, as the α-end angulation increased, the mesial displacement and extrusive tendency of the anchor teeth became more pronounced. The maximum mesial displacement of tooth 24 increased from 5.307 × 10^−3^ mm at 0° to 6.672 × 10^−3^ mm at 30°, while the extrusion increased from 1.593 × 10^−3^ mm to 8.172 × 10^−3^ mm. Overall, the root control of the impacted canine in the TPA group was slightly inferior to that in the MI group. Clinically, higher system stiffness means a higher load-deflection ratio which may produce a strong instantaneous orthodontic force with a shorter duration, resulting in greater force decay during treatment (Mall et al., 2023). Meanwhile, the cumulative effect of repeated force applications on the anchor teeth may lead to occlusal plane canting, which is unfavorable for sustained force delivery.

In TPA group, which used dental anchorage, increasing the α-end angulation produced significant vertical extrusion of maxillary left first premolar, accompanied by a tendency for crown inclination toward the edentulous space. This finding indicates that using teeth as anchorage is unfavorable for space maintenance. However, when the treatment plan involves extraction of the first premolar, this tooth can serve as a temporary anchorage unit and be extracted afterward. Clinically, a TPA or MI is often combined with T-loops to facilitate the eruption of impacted canines. Although temporary anchorage devices (TADs) provide excellent anchorage control, their potential complications—including loosening, fracture, pain, or soft tissue inflammation—remain a concern for some clinicians (Dalessandri et al., 2014). As a moderate anchorage device, the TPA is widely used in clinical practice because of its non-invasive nature. In the present study, appropriate anchorage control in the upper arch was achieved through the combined use of a TPA and posterior teeth during canine retraction. The findings demonstrate that effective distal movement of the maxillary canine can be accomplished regardless of the anchorage system employed. However, the use of TPA inevitably results in mesial displacement and varying degrees of extrusion of the anchor teeth. In clinical practice, precise control of the T-loop angle is often difficult to achieve, and the cumulative effect of repeated force applications on the anchor teeth may lead to occlusal plane canting. Previous studies have shown that an isolated TPA does not provide maximal anchorage in premolar extraction cases when incisors are retracted (Diar-Bakirly et al., 2017; Alhadlaq et al., 2016; Liu et al., 2009). Therefore, if the clinical scenario requires avoiding the use of MI, it may be necessary to consider devices that provide nearly rigid anchorage, such as a tooth-borne hyrax expander.

In the present study, the morphology and position of the T-loop were standardized by placing it close to the canine and constraining its dimensions. However, in clinical reality, the mechanical behavior of a T-loop is influenced by four primary factors: the load–deflection rate of the spring, the location of the activation bends, the magnitude of loop activation, and the position of the archwire relative to the interbracket distance (Hsu et al., 2016). Clinically, in addition to the free wire length having a substantial effect on the force magnitude of a T-loop, its mechanical behavior is also influenced by factors such as the material properties of the archwire, the location of activation bends, and the extent of loop activation (Hsu et al., 2016). The load–deflection rate of the spring is strongly associated with the material properties of the archwire, as well as the geometric configuration and spatial position of the spring. In this study, β-titanium alloy wires were selected for clinical application because it exhibits a flatter load–deflection curve (Menghi et al., 1999). From a mechanical standpoint, the greater the curvature of the force–deflection curve (as observed in stainless steel wires), the greater the force loss per millimeter of deactivation. Conversely, TMA exhibits a flatter force–deflection curve, resulting in substantially less force decay during deactivation—that is, a lower load/deflection (L/D) ratio (Mall et al., 2023). Furthermore, a T-loop fabricated from TMA demonstrates a lower load–deflection rate than an identical loop made from stainless steel (Burstone and Goldberg, 1980; Menghi et al., 1999). Furthermore, research indicates that a taller T-loop with greater apical height produces a higher M/F ratio and lower force delivery per unit of activation (Viecilli and Freitas, 2018). However, in clinical practice, the dimensions and placement of T-loops are frequently adjusted according to patient-specific anatomical constraints, such as the depth of the oral vestibule and overall comfort. During retraction, a T-loop positioned more anteriorly increases the α-moment, whereas a more posterior position enhances the β-moment. In this study, the T-loop was positioned close to the transposed canine to increase the α-moment (Figure 8), thereby facilitating distal root movement of the transposed canine. The horizontal force component retracted the canine, while the vertical positioning of the loop—relative to the bracket on the transposed canine—enabled translation without undesirable extrusion (Hsu et al., 2016). Additionally, a greater inter-bracket distance proved favorable for biologically compatible tooth movement. Regarding the position of the T-loop, a more anterior placement increases the α-moment, whereas a more posterior placement enhances the β-moment, which may serve as a reference for clinical application.

FEA is an appropriate method for evaluating force and stress distribution during the eruption of impacted canines, owing to its advantages of virtual simulation, reproducibility, and the ability to visualize internal stress patterns (Harsha et al., 2023). Previous FEA studies on impacted canines have primarily focused on palatally displaced cases; however, the present study simulated the clinical challenges associated with buccally impacted canines by modeling two different anchorage systems, thereby offering practical implications for clinical orthodontics. Although FEA effectively quantifies force systems and stress distributions during canine traction, it remains inherently a static simulation and cannot account for the biological remodeling processes that occur during actual tooth movement. Future studies employing iterative finite element methods may incorporate preactivated T-loops to simulate long-term stress relaxation within springs and evaluate its influence on tooth displacement, thereby providing deeper insight into the biomechanical mechanisms underlying orthodontic tooth movement. Finite element analysis (FEA) is an appropriate method for evaluating stress distribution during the eruption of impacted canines due to its advantages of reproducibility and visualization (Harsha et al., 2023). However, this study has limitations: both the periodontal ligament and alveolar bone were modeled using linear elastic and static assumptions, which cannot account for the biological remodeling processes that occur during actual tooth movement. Furthermore, the present model was based on a single-case simulation of a 13-year-old child, and there are considerable variations among patients in terms of bone density, root morphology, and impaction position. These mechanical results should be regarded as preliminary evidence. Future studies should further validate and refine the experimental findings through *in vitro* mechanical simulations, and clinical application should be integrated with the patient’s individual biological response and professional judgment.

## Conclusion

Based on the finite element analysis, the following conclusions were drawn: Distal movement of mesially impacted canines can be effectively achieved using either a MI or a TPA as anchorage in combination with a T-loop. The α-end gable bend angle of the T-loop is a critical determinant of the canine displacement pattern. When the α-end angulation was 0°, the tooth exhibited uncontrolled tipping, characterized by distal crown displacement and mesial root displacement. As the angulation increased to 15° and 30°, the difference between crown and root displacement gradually diminished, the center of rotation shifted apically, and the movement pattern transitioned from tipping to translation, indicating significantly enhanced root control. In three dimensions, increasing the gable bend angle generated lingual root torque and an intrusive effect, which helped maintain the root apex within the alveolar bone and reduced the risk of cortical perforation. Comparison of the two anchorage systems revealed that the micro-implant provided absolute anchorage greater anchorage stability. In contrast, the tooth-borne TPA resulted in slightly greater canine displacement under identical force magnitudes; however, this was accompanied by mesial movement and extrusion of the anchor teeth, indicating a certain degree of anchorage loss. Clinically, an α-end gable bend angle of 15°–30° is recommended to achieve effective root control and controlled distal movement of impacted canines. Clinically, incorporating an appropriate α-end gable bend angle may be beneficial for effective root control and controlled distal movement of impacted canines.

## Data Availability

The original contributions presented in the study are included in the article/Supplementary Material, further inquiries can be directed to the corresponding author.
