# Supplementary figures and images for: Finite element analysis of impacted canine disimpaction: effects of anchorage systems and T-loop gable bend angles
Source: Front Bioeng Biotechnol. 2026 Jun 5;14:1850735. doi: 10.3389/fbioe.2026.1850735 (PMC13279527; doi:10.3389/fbioe.2026.1850735)

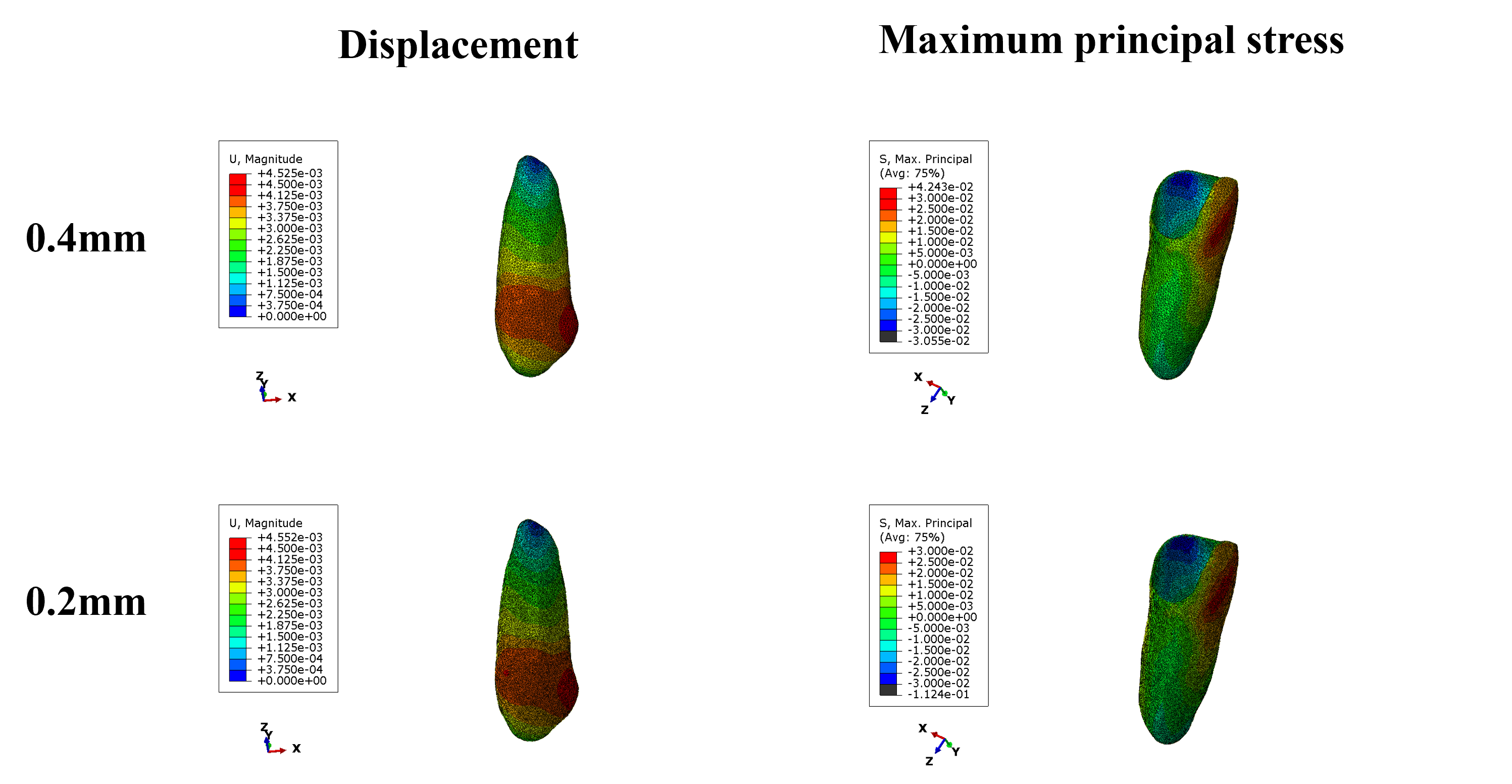

Supplement: Supplementary file 1 [file Image1.tif]
